# A Child with Crohn’s Disease: Problems and Stress Level of Parents–Caregivers—A Cross-Sectional Study

**DOI:** 10.3390/nursrep14010034

**Published:** 2024-02-16

**Authors:** Małgorzata Pasek, Monika Stoietskyi, Anna Goździalska, Małgorzata Jochymek

**Affiliations:** 1 Department of Nursing, Faculty of Health, University of Applied Sciences in Tarnów, 8th Mickiewicz St, 33-100 Tarnów, Poland; malgorzata_pasek@wp.pl; 2 Faculty of Health and Medical Studies, Andrzej Frycz Modrzewski Krakow University, 1st Grudziński St, 30-705 Krakow, Poland; monikabuk@o2.pl (M.S.); mjochymek@afm.edu.pl (M.J.)

**Keywords:** Crohn’s disease, informal caregiver, parent, stress

## Abstract

Crohn’s disease (CD) is a chronic inflammatory bowel disease. The perception of the disease, its troublesome symptoms and the highly involved treatment mean that living with CD requires not only the affected child having to learn to cope with it, but also their parents. The aim of the study was to examine the problems and levels of stress in parents–caregivers of children with CD. The study was cross-sectional and conducted using the diagnostic survey method. An original questionnaire was used to assess the socio-demographic situation and problems of caring for a child with CD, and the standardised PSS-10 questionnaire was added. The study group consisted of 60 parents who accompanied their children during hospitalization. The surveyed caregivers of children with CD found it difficult to maintain a specialised diet and deal with the need for hospitalization and the chronic use of medications. Access to the necessary knowledge about the disease posed the least difficulty in everyday life for a child with CD. The vast majority of parents (50, 83.34%) felt a high level of stress related to caring for a child with CD and, simultaneously, a lack of acceptance of the child’s disease (39, 65%). The age of the respondents did not have a statistically significant effect on the occurrence of problems related to everyday functioning. The acceptance of the child’s disease by the parents and informal caregivers of children with CD reduced their stress level. Parents could count on support from their relatives but, unfortunately, institutional support was not properly provided.

## 1. Introduction

Crohn’s disease (CD) is a chronic disease and patients have to bear its consequences, including socio-economic ones, for the rest of their lives. In the contemporary literature, much attention is paid to the treatment of CD, alleviating symptoms and improving the quality of life. However, parents of paediatric patients are often in the background. Studies of dyads of affected people and their so-called caregivers indicate that both the caregiver and the patient experience the disease to the same extent [1].

The exact etiopathogenesis of CD is still unknown. In addition to genetic factors, the environment also has a significant impact on the development of the disease [2]. Several genes have been studied for CD, but significant associations have so far been identified with *the NOD2, IL23R* and *ATG16L1* genes. In CD, the *NOD2*/*CARD15* gene (*IBD1* locus on chromosome 16) is associated with ileal involvement, fibrostenotic disease, an earlier age of onset, and a family history of CD [3]. If a mutation of this gene is present, the potential for developing the disease increases by as much as 20–40 times [4]. The risk of contracting CD also increases if someone in the patient’s immediate family has the disease [5]. Factors predisposing to CD also include smoking, a diet low in fibre and rich in carbohydrates, an altered microbiome, as well as taking antibiotics, oral contraceptives and non-steroidal anti-inflammatory drugs [3]. During the analysis of environmental factors, an increase in morbidity was observed in countries with highly developed industries [6].

Intestinal symptoms account for approximately 80% of the clinical manifestations of CD. Common extraintestinal manifestations (EIMs) of CD include large joint inflammation, uveitis, iritis, episcleritis, erythema nodosum, and pyoderma gangrenosum [7]. The EIMs of the disease also include the development of inflammatory changes in the bile ducts, liver, eye and vascular system [8].

The clinical picture in CD consists of a number of symptoms [9]. General symptoms include weakness of the body, increased temperature and weight loss. There may be local symptoms which depend on the location of the inflammatory changes [10]. CD is typically characterised by transmural enteritis and can affect any part of the digestive tract from the mouth to the anus. About 25% of patients have colitis only, 25% ileitis only, and 50% ileocolonic. In approximately 5–15% of patients, the oral cavity or gastroduodenal involvement is also present [3].

CD is characterised by exacerbation and remission phases. According to Zagórowicz, the exacerbation phase occurs constantly in about 10–15% of patients [11]. The persistence of remission enables the healing of inflammatory changes in the digestive tract. In most patients, the course of the disease is described as severe and often aggressive, with fragments of the gastrointestinal tract destroyed by the formation of strictures and fistulas. As a consequence, surgical treatment is included [12]. In these patients, complex treatment is initiated, including biological and immunosuppressive medications [13]. Medications used to maintain remission include 5-aminosalicylic acid products, immunomodulators (azathioprine, 6-mercaptopurine, methotrexate) and tumour necrosis factor inhibitors (infliximab, adalimumab, certolizumab and golimumab). Surgical interventions, such as bowel resection, stricturoplasty or abscess drainage, are required in up to two-thirds of CD patients during their lifetime [3]. In about 40% of patients, the course of the disease is mild and the remission phase predominates. However, 10–20% do not require treatment to maintain remission [14].

The treatment of patients with CD includes two forms: nutritional and pharmacological [15]. The first involves adjusting the type of meals consumed due to the occurrence of pain and diarrhoea, and additionally a lack of appetite. The nutritional status is negatively affected by impaired absorption, as well as higher energy demand due to the increased intensity of catabolic reactions [7]. Nutritional recommendations are individual, depending on the needs. An elimination diet is usually used, and the observation of assimilated foods is crucial here. The method of treatment depends on the patient’s condition after diagnosis and the initiation of therapy. Sometime it takes many weeks of nutritional treatment before the body improves [16]. Pharmacological treatment involves the systematic administration of medications according to an individual therapy scheme [15].

The perception of the disease described above, its troublesome symptoms and a highly involved treatment mean that living with CD requires that both a small patient and their parents have to learn how to cope with the disease, exacerbations and ailments. It also requires the ability to introduce CD into normal everyday functioning. It is a difficult process associated with a sense of difference, particularly in children. The burden on parents and sometimes their inability to improve their child’s condition may also affect the daily struggle of the small patient with this chronic disease [17]. In addition, caring for a chronically-ill child may also have a negative impact on the physical and mental functioning of the caregiver if they are not provided with the appropriate support [18].

The aim of the study was to examine and describe the problems and levels of stress in parents–caregivers of children with CD.

## 2. Materials and Methods

The research project was cross-sectional and conducted using the diagnostic survey method and the questionnaire technique. To achieve the aim of the study, an original questionnaire was developed to assess the socio-demographic situation and problems of caring for a child with CD, and the standardised PSS-10 questionnaire (Perceived Stress Scale) was added. The internal consistency of the PSS-10 is 0.86 (Cronbach’s alpha). The standardised PSS-10 questionnaire is used to measure perceived stress. It consists of 10 questions concerning subjective feelings related to personal events, problems and behaviours, as well as ways of dealing with them. The scale assesses the intensity of stress occurring in the last month. There are five answer options for each question, for which the responses receive the following scores: never—0, almost never—1, sometimes—2, quite often—3, and very often—4. It is possible to obtain a maximum score of 40. The results are converted into standardisation units determined using the Sten scale and mean: a score of 1–4: low stress; 5–6: moderate stress; and 7–10: high stress [19].

The study was conducted in accordance with the Declaration of Helsinki.

The criteria for inclusion in the study were as follows: informed consent, the presence of CD in a child, and a mental state making it possible to understand and complete the questionnaire independently. The exclusion criteria were: undiagnosed CD, a lack of consent of the examined parent, and a mental state that made it impossible to understand and complete the questionnaire independently.

The study was conducted from February to April 2021 in a paediatric hospital in Krakow, Poland.

The questionnaires were given to parents in paper form. Parents were informed about the researchers’ compliance with the regulation on the protection of personal data. A total of 70 questionnaires were distributed to parents who had been previously informed about the purpose of the study, voluntary participation in the project and anonymity. All gave informed consent to participate in the study. All in all, 60 fully completed questionnaires were returned, becoming the basis for the calculations.

Statistical analysis was performed using the SPSS 20 software. The *t*-test for independent variables, the one-way analysis of variance and Spearman’s rho correlation coefficient were used in the study. The selection of parametric or non-parametric methods was preceded by checking the normality of variable distributions with the Kolmogorov–Smirnov test. A significance level of *p* < 0.05 was assumed.

Table 1 shows the characteristics of the studied group.

The average age of the surveyed parents was 41.72 ± 4.73 years; the youngest person was 34 years old and the oldest was 53.

Among the working people, over one-third of the respondents (21, 37.5%) worked remotely, and the rest (35, 62.5%) worked on-site. Only two respondents practiced a medical profession, and the rest (nearly 92%) represented other professions.

## 3. Results

The study involved 60 parents and the vast majority (58, 96.67%) had one child with CD. Two had more than one child with this diagnosis.

Table 2 shows the socio-demographic characteristics of the children whose parents participated in the study.

The average age of children at the time of diagnosis was 9.13 ± 3.01 years and ranged from 2 to 15 years. The average duration of CD in a child on the day of completing the questionnaire was 4.05 ± 2.65 years and ranged from 1 to 12 years.

**Figure 1:**
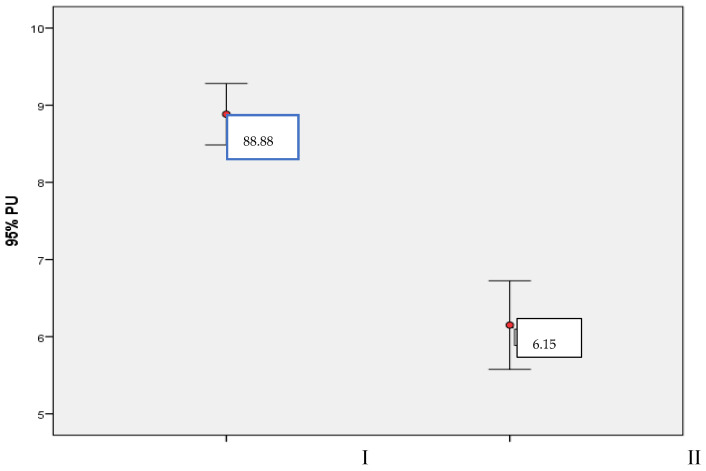
Stress level after a child’s disease was diagnosed and current stress level (scores of 0–10). I—stress level after a child’s disease was diagnosed (scores of 0–10), II—current stress level (scores of 0–10).

The average level of stress in parents after learning the child’s diagnosis was a score of 8.88 ± 1.54 and the scores ranged from 2 to 10. The currently perceived level of stress in parents due to the child’s condition was a score of 6.15 ± 2.22 and the scores ranged from 1 to 10 on a scale of 0–10 (Figure 1).

The level of stress in respondents may result from the occurrence of problems related to their child’s disease. The greatest difficulties in everyday life with a child with CD concerned the adverse effects of treatment (score = 4.07) and obtaining support from social support institutions (score = 4.02). Another major difficulty was maintaining a specialised diet (score = 3.82), the need for hospitalisation (score = 3.75) and the chronic use of medications (score = 3.63). The smallest difficulty in everyday life with a child with CD was access to the necessary knowledge about the disease (score = 2.25) and enrolling the child in kindergarten/school (score = 1.97). The results of the study show that most parents had problems with accepting their child’s disease (39, 65%). The vast majority of respondents indicated, however, that the help they received from relatives and medical personnel was properly provided. However, the majority claimed (43, 71.6%) that the support offered through social support institutions was insufficient. Most parents (42, 70%) declared they had adequate access to the necessary knowledge about the disease. The majority of respondents (36, 60%) also had no major problems with observing the vaccination schedule for their children. However, the perception of problems related to respiratory and digestive tract infections was distributed differently. Table 3 shows all the above aspects of the assessment.

The subject of the study was also the surveyed parents’ assessment of problems occurring in their children. For 55% of the respondents, the acceptance of the disease by the child was somewhat a problem (18, 30.0%) or definitely a problem (15, 25.0%). Table 4 shows this and the other aspects examined.

Our study showed that the greatest problem for children, in the opinion of the surveyed parents, was the side effects of medications (48, 80% of the respondents), the related chronic use of medications (41, 68.3%) and minimising the remaining symptoms of the disease (40, 66.6%). Contact with peers was rated as definitely not (10, 16.7%) or not much of a problem (41, 68.3%) for children.

The answer ‘I do not know’ was rarely or never given, although it was in the case of, for example, acceptance of the disease by the child and the parent, pain relief and the child’s contact with peers. This may indicate the awareness of and strong opinions on the above-mentioned topics, as presented in Table 3 and Table 4.

The study shows (Table 5) that the age of the respondents did not have a statistically significant effect on the occurrence of problems related to everyday functioning as indicated by the parents of children with CD (*p* > 0.05).

The study shows (Table 6) that the respondents indicated they had greater difficulties in maintaining a specialised diet in nutritional treatment with the use of a gastric/intestinal tube (score = 4.55; *p* < 0.0001) and greater difficulties with the physical activity of their children (score = 3.94; *p* = 0.0019) compared to parents of children who did not receive this form of treatment.

Based on the standardised PSS-10 questionnaire, the level of stress of the surveyed parents was determined, referring to their feelings from the last month. It was shown that as many as 83.34% (50) of parents felt stress related to caring for a child with CD at a high level. The rest, that is, five respondents (8.33%), indicated an average level of stress and five respondents (8.33%) a low level of stress.

It was shown that parents who accepted their child’s disease to a lesser extent had a higher level of current stress (rho = 0.428) and a higher objective level of stress (rho = 0.380). Parents who had more difficulties in receiving help from relatives had an increased level of stress at the time of diagnosis (rho = 0.281), currently (rho = 0.531) and objectively (rho = 0.516). Table 7 shows the details.

## 4. Discussion

CD is a chronic disease that significantly affects everyday life, as it deteriorates the quality of life and causes numerous difficulties and worries for both patients and their caregivers [20]. Therefore, studying the problems faced by the parents of children with CD is extremely important because both parents and their children must struggle with the disease every day for the rest of their lives. Parents’ problems often take a back seat, while how they deal with their child’s disease has a very significant impact on the quality of life of their children and the course of the disease. Worries and caring for ill children cause feelings of stress, guilt and frustration. Both patients and their caregivers express the need to talk about their fears and make them visible, recognised and understood [1]. It has also been proven that the caregivers’ burden remains high even if the patient is in clinical remission [18]. In addition, they have a strong and partially unsatisfied need to express their fears and relieve the negative emotions towards caregivers [20]. It is therefore very important for medical personnel to notice the existing situation and counteract it through supporting and educating the parents–caregivers of children with CD. In addition, society should be made aware of the problems faced by parents and how help and support can be provided [21]. It is also worth noting the need to develop social support institutions, which, among other things, can help with financial support for the family [22]. A phenomenon being observed more and more often is the creation of support groups, where parents of children with CD can share their experiences and valuable practical comments, as well as discussing problems, receiving mutual support and getting help [23]. This has a positive effect on reducing their stress.

Pharmacotherapy most often includes the use of biological therapy, steroid therapy and immunosuppressive treatment [24]. It requires strict application of the recommended doses and also causes a number of side effects [25]. In our study, the biggest concern of parents was the side effects of the chronic use of medications by children. However, the potential benefits of the applied treatment outweigh the side effects that affect the child’s health to a greater or lesser extent.

Parents of paediatric patients undergoing nutritional treatment with the use of a gastric/intestinal tube indicated greater difficulties in adhering to a specialised diet and their child undertaking physical activity, compared to parents of children not undergoing such treatment. Nutritional therapy lasts several weeks, depending on the individual factors for each paediatric patient [12]. During this time, the risk of rejection of an ill child by a group of peers increases, which in turn may affect the child’s mental condition [26]. Our study showed that parents did not perceive their ill child’s contact with peers to be a problem. For parents of children undergoing biological treatment, the greatest problem is the need for regular and relatively frequent hospitalisations. Therapy forces parents to divide their time between work and caring for a chronically-ill child, while for a child it may mean the inability to participate in school and everyday activities [3,27]. Our study confirms that the hospitalisation of a child was a big problem for parents–caregivers.

Constant stress is an extremely important problem [17,28]. Parents who showed a lower degree of acceptance of their child’s disease had a higher level of stress related to the everyday problems associated with caring for an ill child. The subjective assessment of parents regarding the level of stress correlated with the results obtained in the PSS-10 stress level questionnaire. The high level of stress, together with the inability to cope with it, had a negative impact on the mental and physical health of the parents, and indirectly on the health of the child. Thus, following Larsson et al., attention should be paid to the important role of both instrumental and emotional support, for which parents ask, particularly at the time of diagnosis and exacerbation of the disease [21]. Unfortunately, observing the current state of psychological care in Poland, it can be concluded this is insufficient. Therefore, the priority action of the medical personnel taking care of a child with CD and their parents is to improve the mental state of the parents by providing professional services. They should pay particular attention not only to incisively and empathetically providing information about the child’s disease but also demonstrating understanding of the parents’ situation [17]. The acceptance of the disease by caregivers allows them to adopt a task-oriented attitude focused on activities that parents can perform to improve the quality of life for both them and their child. In our study, as many as 65% of parents had a problem accepting their child’s disease, and men showed less acceptance [20,27].

The research results obtained were significantly influenced by the parents’ life situation at a given moment, as well as their individual ways of coping with problems in everyday life. The duration of the child’s disease also had an impact on the period during which the parents adapted to the new life situation. The actual course of the child’s disease also plays an important role in perceiving a given aspect as a problem and its severity. Coping with the disease would be easier if appropriate institutions properly provided social support, the lack of which, as demonstrated in this study, results in a significant increase in the level of stress in the parents of children with CD.

### Limitations

Our study was cross-sectional. In addition, it was conducted during the SARS-CoV-2 pandemic, which made it significantly more difficult to obtain a larger group of respondents. The undertaking of a longitudinal study, taking into account the duration of the disease, emerging side effects and family relationships, could more broadly present the problems of caregivers in caring for a child with CD.

## 5. Conclusions

A great difficulty for the surveyed caregivers of children with CD was maintaining a specialised diet, the need for hospitalisation and chronic use of medications.

Access to the necessary knowledge about the disease posed the least difficulty for everyday life with a child with CD.

Acceptance of the child’s disease by the caregivers–parents of children with CD reduced their stress levels.

Parents could count on support from their relatives but, unfortunately, institutional support was not properly provided, so we suggest defining the new guidelines to improve communication policy.

## Figures and Tables

**Table 1:** Socio-demographic characteristics of the studied group.

Variables		*N* Respondents	% of Respondents
Gender	female	41	68.33
	male	19	31.67
Age	up to 40 years	26	43.33
	more than 40 years	34	56.67
Marital status	single	1	1.67
	married	38	63.33
	widowed	4	6.67
	divorced	9	15.00
	in an informal relationship	8	13.33
Children	one	11	18.33
	two	24	40.00
	three	14	23.33
	four	10	16.67
	five or more	5	8.33
Education	middle secondary	1	1.66
	occupational	15	25.00
	secondary	22	36.67
	higher	22	36.67
Place of residence	city	36	60.00
	village	24	40.00
Occupational status	contract of employment	35	58.33
	civil law contract	10	16.67
	self-employment	11	18.33
	unemployment benefit	4	6.67

**Table 2:** Socio-demographic and clinical characteristics of the children with CD.

Variables		*N* Respondents	% of Respondents
the age at which the disease was diagnosed	up to 10 years	32	53.33
	more than 10 years	28	46.67
the period since diagnosis	up to 5 years	39	65.00
	more than 5 years	21	35.00
nutritional treatment with a gastric/intestinal tube	yes	31	51.67
	no	29	48.33
biological therapy	yes	27	45.00
	no	33	55.00

**Table 3:** Parents’ problems related to caring for a child with CD.

Variables	Definitely Not a Problem		Not Much of a Problem		I Do not Know		Somewhat a Problem		Definitely a Problem		Mean	SD
	*N*	%	*N*	%	*N*	%	*N*	%	N	%	$\overset{-}{x}$	σ
parental acceptance of the disease	3	5.00	18	30.0	0	0.0	25	41.7	14	23.3	3.48	1.28
help received from relatives (partner, family, friends)	14	23.3	22	36.7	1	1.7	7	11.7	16	26.7	2.82	1.58
help and support from medical personnel	8	13.3	31	51.7	5	8.3	15	25.0	1	1.7	2.50	1.07
help from social support institutions	2	3.3	7	11.7	8	13.3	14	23.3	29	48.3	4.02	1.19
family financial situation	6	10.0	20	33.3	0	0.0	15	25.0	19	31.7	3.4	1.48
access to the necessary knowledge about the disease	16	26.7	26	43.3	5	8.3	13	21.7	0	0.0	2.25	1.08
the need for the child to be hospitalised	4	6.7	12	20.0	0	0.0	23	38.3	21	35.0	3.75	1.31
avoiding infections of the child’s respiratory and digestive tracts	6	10.0	24	40.0	9	15.0	19	31.7	2	3.3	2.78	1.11
following a specialised diet	3	5.0	14	23.3	1	1.7	15	25.0	27	45.0	3.82	1.36
preventing respiratory and gastrointestinal infections in the child	6	10.0	24	40.0	9	15.0	19	31.7	2	3.3	2.78	1.11
enrolling a child in kindergarten/school	16	26.7	36	60.0	2	3.3	6	10.0	0	0.0	1.97	0.84
observing the vaccination schedule	5	8.3	31	51.7	6	10.0	16	26.7	2	3.3	2.65	1.07

**Table 4:** Problems occurring in a child with CD according to the surveyed parents.

Variables	Definitely Not a Problem		Not Much of a Problem		I Do not Know		Somewhat a Problem		Definitely a Problem		Mean	SD
	N	%	N	%	N	%	N	%	N	%	$\overset{-}{x}$	σ
acceptance of the disease by the child	0	0.0	27	4	0	0.0	18	30.0	15	25.0	3.35	1.29
relieving the child’s pain	4	6.7	24	40.0	0	0.0	27	45.0	5	8.3	3.08	1.21
minimising the remaining symptoms of the disease	1	1.7	17	28.3	2	3.3	32	53.3	8	13.3	3.48	1.10
chronic use of medications	4	6.7	12	20.0	3	5.0	24	40.0	17	28.3	3.63	1.28
side effects of the treatment	0	0.0	11	18.3	1	1.7	21	35.0	27	45.0	4.07	1.10
child’s contact with peers	10	16.7	41	68.3	0	0.0	9	15.0	0	0.0	2.13	0.87
child’s physical activity	3	5.0	13	21.7	1	1.7	36	60.0	7	11.7	3.52	1.11

**Table 5:** Relationship between the occurrence of problems related to the child’s disease and the age of the examined parents.

Variables	Up to 40 Years		More than 40 Years		*p*
	Mean	SD	Mean	SD	
acceptance of the disease by the examined parent	3.19	1.27	3.71	1.27	0.1252
help received from relatives (partner, family, friends)	2.69	1.54	2.91	1.62	0.5978
help and support from medical personnel	2.35	0.98	2.62	1.13	0.3324
help from social support institutions	3.92	1.13	4.09	1.24	0.5973
family’s financial situation	3.08	1.49	3.56	1.44	0.2113
relieving the child’s pain	3.04	1.28	3.12	1.17	0.8043
minimising the remaining symptoms of the disease	3.50	1.21	3.47	1.02	0.9191
access to the necessary knowledge about the disease	2.27	1.08	2.24	1.10	0.9055
the need for hospitalisation	3.77	1.27	3.74	1.36	0.9218
chronic use of medications	3.81	1.23	3.50	1.31	0.3588
side effects of the treatment	4.35	1.02	3.85	1.13	0.0860
avoiding infections of the respiratory and digestive tracts of the child	2.69	1.01	2.85	1.18	0.5816
following a specialised diet	3.81	1.41	3.82	1.34	0.9648
acceptance of the disease by the child	3.12	1.28	3.53	1.28	0.2196
enrolling a child in kindergarten/school	1.73	0.45	2.15	1.02	0.0573
observing the vaccination schedule	2.50	0.99	2.76	1.13	0.3470
child’s contact with peers	2.19	0.85	2.09	0.90	0.6510
child’s physical activity	3.62	1.17	3.44	1.08	0.5522

**Table 6:** Relationship between a specialist diet and the physical activity of a child and the child being treated with nutritional preparations through a gastric/intestinal tube.

Treatment through a Gastric/Intestinal Tube	Parameter	Following a Specialised Diet	Child’s Physical Activity
Yes	Mean	4.55	3.94
	SD	0.93	0.85
No	Mean	3.03	3.07
	SD	1.32	1.19
In total	Mean	3.82	3.52
	SD	1.36	1.11
*p* (the level of significance)		0.0000	0.0019

**Table 7:** Relationship between the respondents’ level of stress, acceptance of the disease and the support received.

Variables according to the PSS-10 Scale		Acceptance of the Child’s Disease by the Parent(s)	Support Received from Relatives (Partner, Family, Friends)
stress level after diagnosing a child [scores of 0–10]	rho	0.175	0.281
	*p*	0.1811	0.0294
current stress level [scores of 0–10]	rho	0.428	0.531
	*p*	0.0006	0.0000
overall stress level [scores of 0–40]	rho	0.380	0.516
	*p*	0.0028	0.0000

## Data Availability

The data presented in this study are available on request from the corresponding author.

## Public Involvement Statement

No public involvement in any aspect of this research.

## Guidelines and Standards Statement

This manuscript was drafted against the STROBE for cross-sectional studies.
